# Glass bead system to study mycotoxin production of *Aspergillus* spp. on corn and rice starches

**DOI:** 10.1007/s00253-024-13190-7

**Published:** 2024-05-29

**Authors:** Katalin Inotai, Ildikó Bata-Vidács, Ákos Tóth, Judit Kosztik, Mónika Varga, András Szekeres, István Nagy, István Nagy, Csaba Dobolyi, Mária Mörtl, András Székács, József Kukolya

**Affiliations:** 1 Agro-Environmental Research Centre, Institute of Environmental Sciences, Hungarian University of Agriculture and Life Sciences, Páter Károly u. 1, 2100 Gödöllő, Hungary; 2 Food and Wine Research Institute, Eszterházy Károly Catholic University, Leányka u. 6, 3300 Eger, Hungary; 3 HUN-REN-EKKE Lendület Environmental Microbiome Research Group, Eszterházy Károly Catholic University, Leányka u. 6, 3300 Eger, Hungary; 4 Heart and Vascular Center, Semmelweis University, Városmajor u. 68, 1085 Budapest, Hungary; 5 Department of Microbiology, Faculty of Science and Informatics, University of Szeged, Közép fasor 52, 6726 Szeged, Hungary; 6 Seqomics Biotechnology Ltd., Vállalkozók útja 7, 6782 Mórahalom, Hungary; 7 Institute of Biochemistry, Biological Research Centre, HUN-REN, Temesvári krt. 62, 6726 Szeged, Hungary; 8 Department of Environmental Safety, Institute of Aquaculture and Environmental Safety, Hungarian University of Agriculture and Life Sciences, Páter Károly u. 1, 2100 Gödöllő, Hungary

**Keywords:** Sterigmatocystin, Aflatoxin B_1_, *Aspergillus creber*, *Aspergillus flavus*, Corn starch, Rice starch

## Abstract

**Abstract:**

Mycotoxin production by aflatoxin B1 (AFB1) -producing *Aspergillus flavus* Zt41 and sterigmatocystin (ST) -hyperproducer *Aspergillus creber* 2663 mold strains on corn and rice starch, both of high purity and nearly identical amylose-amylopectin composition, as the only source of carbon, was studied. Scanning electron microscopy revealed average starch particle sizes of 4.54 ± 0.635 µm and 10.9 ± 2.78 µm, corresponding to surface area to volume ratios of 127 1/µm for rice starch and 0.49 1/µm for corn starch. Thus, a 2.5-fold difference in particle size correlated to a larger, 259-fold difference in surface area. To allow starch, a water-absorbing powder, to be used as a sole food source for *Aspergillus* strains, a special glass bead system was applied. AFB1 production of *A. flavus* Zt41 was determined to be 437.6 ± 128.4 ng/g and 90.0 ± 44.8 ng/g on rice and corn starch, respectively, while corresponding ST production levels by *A. creber* 2663 were 72.8 ± 10.0 µg/g and 26.8 ± 11.6 µg/g, indicating 3–fivefold higher mycotoxin levels on rice starch than on corn starch as sole carbon and energy sources.

**Key points:**

• *A glass bead system ensuring the flow of air when studying powders was developed*.

• *AFB1 and ST production of A. flavus and A. creber on rice and corn starches were studied*.

• *3–fivefold higher mycotoxin levels on rice starch than on corn starch were detected*.

## Introduction

Aflatoxins (AFs), though natural substances, are among the presently known most carcinogenic compounds. These most important mycotoxins are produced mainly by numerous species from the genus *Aspergillus* (Rank et al. 2011). These fungi can contaminate cereals before harvest on the fields or during storage in storehouses, resulting in substantial economic losses throughout the world (Wilkinson et al. 2004). Aflatoxins can cause acute hepatic failure in humans, as well as in higher vertebrates and poultry (Wogan 1992; Hua et al. 2020).

Sterigmatocystin (ST) is a precursor of AFs in their biosynthesis, first isolated from *Aspergillus versicolor* in 1954 (Soriano del Castillo 2007). ST is also toxic, mutagenic, and carcinogenic (Kövesi et al. 2021; Zhou et al. 2023; Zingales et al. 2020), although less than aflatoxin B_1_ (AFB1) (Alonso-Jauregui et al. 2023). The oral LD_50_ values of ST and AFB1 for male rats are 60–800 and 5.5 mg/kg body weight, respectively (Tabata 2011). ST is produced by some *Aspergillus* species like *A. versicolor*, *A. nidulans*, and *A. sydowii*, and also by some species of *Bipolaris*. The major ST-producer among them is *A. versicolor* (Tabata 2011; Mahata et al. 2022).

According to dozens of studies in the scientific literature (Yu 2012; Schmidt-Heydt et al. 2009, 2010; Marroquin-Cardona et al. 2014; Dövényi-Nagy et al. 2020), the optimal a_w_ for mycotoxin production is at the range of 0.92–0.96 a_w_, at 27–35 °C. Lv et al. (2019) found that the highest level of AFB1 was produced by *Aspergillus flavus* on rice at 0.96 a_w_ and 33 °C after 2 weeks. Bernaldez et al. (2017) studied mycotoxin production on corn substance, and found that the maximum AFB1 production was at 30 °C and 0.98 a_w_ (Cotty 1988). In the studies of Casquete et al. (2017), the maximum AFB1 production by *A. flavus* strains occurred at pH 5.0.

It is well known that the carbon source has a significant impact on AF formation. Simple sugars like maltose or glucose, formed from starches, support AF production (Abdollahi and Buchanan 1981; Buchanan and Lewis 1984; Luchese and Harrigan 1993; Payne and Brown 1998). A relationship between the activity of alpha-amylase and AF production by *A. flavus* was also reported (Woloshuk et al. 1997). Molds have adapted during their evolution to use starch as carbon and energy sources. *Aspergillus* strains are highly efficient producers of many extracellular polysaccharide-decomposing enzymes (Mojsov 2016; Hu et al. 2011). These strains are used commercially for the production of amylases, which are in turn used in the starch industry to produce sugars from starch (Van der Maarel et al. 2002). Starch consists of two types of molecules: amylose (linear polymer of D-glucose linked by 1,4 glycosidic linkages) and amylopectin (branched polymer of α-D-glucose units with 1,4–1,6 glycosidic linkages) (Suzuki and Suzuki 2021).

The digestibility of rice starches depends on many factors such as the ratio of amylose and amylopectin (Björck 1996), the crystallinity degree (Chung et al. 2006), and the amylopectin’s molecular structure (Srichuwong and Jane 2007). The digestibility is not related to the percentage of amylopectin, but rather to the size of the side chains (degree of polymerization—DP) that make up the molecule, which for longer chains (DP > 37) results in crystalline cores, making enzymatic hydrolysis more difficult. Better digestibility of amylopectin with short side chains (DP = 6–12) has been reported in several publications (Jane et al. 1997; Magallanes-Cruz et al. 2017). The size and shape of the starch particles are also responsible for the digestibility of starch, as more water is adsorbed on the higher surface area of small starch granules promoting enzymatic digestion.

For toxicology studies, it is essential to perform animal feeding trials with higher-than-normal toxin concentrations. For this purpose, the best way to produce toxins is the inoculation of a substrate with the toxin-producing molds under laboratory conditions. Synthetic media support minimal toxin production (1 to 60 µg AFB1 per g medium), whereas maximum yields (700 to 900 µg AFB1 per g medium) could be observed on autoclaved wheat, rice, cottonseed, and corn (Detroy et al. 1971). On solid rice substrate, more than 1 mg/g AFB1 production was obtained in 5 days at 28 °C (Shotwell et al. 1966). Another favored substrate for toxin production is corn grit. According to Epstein et al. (1970), *A. flavus* produced 72 µg/g AFs on corn at 28 °C after 2 days. Winn and Lane (1978) found 35 µg/g AFB1 on cracked corn at 25 °C and 70 µg/g at 30 °C. These data suggest that the AF yield of aspergilli on rice is around ten-fold higher than that on corn.

As for the production of ST on corn or rice substrate, only limited data are available. Lepom and Kloss (1988) tested nineteen *A. versicolor* strains for their production of ST. All isolates were able to produce ST at different levels on a cracked corn substrate, and 53% of the isolates produced more than 500 µg/g of ST. *A. nidulans* produced only 10.4 µg/g ST, while obtained yields for *A. versicolor* 22333, 22332, 22334, and flour-mill isolate 2380 were 186.2, 157.4, 9.3, and 12.3 µg/g, respectively, while the best ST producer strain was flour-mill isolate *A. creber* 2663, with 277.1 µg/g ST production on corn grit (Dobolyi et al. 2021). ST production on rice substrate is submitted by Hajjar et al. (1989), who found that *A. nidulans* produced 4.6–32.6 µg/g rice, although *A. nidulans* is not the best ST-producing *Aspergillus* sp. (Dobolyi et al. 2021). The amounts of the ST produced by *A. creber* on rice reached 100–150 µg/g at pH levels around 6 at 30 °C and up to 400 µg/g at the optimal temperature of 26 °C (non-published data).

As seen in the scientific literature, there is approximately a tenfold yield difference between corn and rice substrates for AF and ST production, although yields strongly depend on mold species, strains, and environmental conditions of production. During our preliminary experiments, we also established that the AFB1 and ST-producing mold strains grown on rice produce larger amounts of mycotoxins than in the case of corn. For animal feeding trials, 7.59 µg/g ST concentration could be obtained with *A. creber* in larger quantities of corn grit (Balogh et al. 2019), while on rice 84.39 µg/g ST could be achieved (non-published data), and the ten-fold difference could be observed under the same conditions with the same mold strain.

Our studies aimed to find the reason behind the phenomenon that mycotoxin production by aspergilli on the rice substrate is much higher than on corn. For this purpose, we used our recently isolated AFB1 and ST-producing *Aspergillus* strains (*A. flavus* Zt41 and the first extreme ST-producer *A. creber* 2663 strains in Hungary (Dobolyi et al. 2013, 2021)) with high toxin production ability. To eliminate the complex organic substrates present in corn and rice grains, high-purity corn and rice starches were used as sole carbon and energy sources. Therefore, it was not necessary to include complex purification of the extract, e.g., by liquid–liquid separation using centrifugal partition chromatography (Endre et al. 2019) or application of more selective detection mode, e.g., high-resolution mass spectrometry in the determination of target compounds. In the choice of culture conditions, the objective was to create appropriate environmental conditions for toxin production, to design a culturing system that best mimics the conditions under which molds grow on corn and rice grains.

Liu et al. (2016) investigated the factors that influence the accumulation of AFB1 in seeds. A strong relationship between nutrients and AFB1 production was found in different cereal grains. Different nutrients have different effects on *A. flavus* growth and mycotoxin production. In the case of lipid-free seeds, AFB1 production significantly decreased. In addition to lipids, other nutrients in the substrate also play a pivotal role in AFB1 biosynthesis and mycelium growth (Liu et al. 2016). Glass beads as a solid support have shown utility not only in plating cell cultures and colony growth (Worthington et al. 2001; Prusokas et al. 2021), but also in the cultivation of filamentous fungi (Bottcher and Conn, 1942; Nguyen et al. 2005; Droce et al. 2013; Ali et al. 2016) showing advantages to both agar plate cultures and liquid cultures.

Taking these aspects into account, the paper fills a notable gap in the literature, particularly concerning the differential production of aflatoxin and sterigmatocystin by *Aspergillus* species on corn and rice substrates. To this aim a novel approach has been taken by culturing aflatoxin and sterigmatocystin-producing strains of *Aspergillus* on glass beads coated with corn and rice starches, simulating the natural growth environment of molds on grains. By applying this novel system, the effect of other compounds and nutrients (for example, soluble sugars, lipids, amino acids, etc.) than starch in the seeds on mycotoxin production can be eliminated.

## Materials and methods

### Chemicals

Corn starch and rice starch used for the AF and ST production studies were purchased from Sigma-Aldrich (Merck Life Science Kft., Budapest, Hungary). The ratio of amylose to amylopectin in corn starch is 27:73 w/w% (Sigma), while rice contains slightly less, 23% amylose (https://www.sigmaaldrich.com/HU/en/product/sial/s4126). All chemicals used in the study were of analytical grade.

### Experimental strains

*A. flavus* Zt41 (NCAIM F.01021) with good AFB1-producing ability was obtained from corn (2009, Baranya County, Hungary); *A. creber* 2663 (NCAIM F.01020) was isolated from a flour mill in Hungary in 2016 and has an outstanding ST-producing capability (Dobolyi et al. 2021). The strains were stored at − 80 °C in 20% glycerol until use.

To prepare the inoculum for the experiments, the mold strains were spread onto PDA (Potato Dextrose Agar, VWR) plates. The plates were incubated at room temperature for 7 days, in the dark. Suspensions of molds were prepared with sterile water with a Potter homogenizer. The final concentrations were set to 10^9^ conidiospore/mL.

### Sterigmatocystin production of *A. creber* 2663 and aflatoxin B_1_ production of *A. flavus* Zt41 on corn and rice starches mounted on glass beads

Into 100 mL glass flasks with screw-tops, 40 g of glass beads with diameters of 2 mm were placed and sterilized by autoclaving. To the flasks, 5 g of rice or corn starch and 3 mL of minimal medium ((NH_4_)_2_SO_4_ 1.25 g, KH_2_PO_4_ 0.5 g, MgSO_4_ × 7 H_2_O 0.5 g in 1000 mL distilled water, pH = 4.8) sterilized by filtration were added, and were mixed with a sterile spoon until the starch suspension covered the glass beads evenly.

The pH was 6.0 at the beginning of the experiment, and each setup was sampled periodically during the 3-week incubation. The fifth samples were used for the determination of pH at the end of the cultivation period. Distilled water was added to the flasks and the final pH values were measured as 4.85 and 5.20 for *A. creber* on rice and corn starch, respectively, and 5.64 and 5.56 for *A. flavus* on rice and corn starch, respectively.

Then, 500 µl of *A. creber* 2663 or *A. flavus* Zt41 suspension prepared as described before was pipetted into each flask in 5–5 parallels, respectively. The flasks were incubated at 26 °C in the dark for 3 weeks. The conditions of cultivation and mycotoxin production were set according to preliminary experiments for mycotoxin production optimisation (non-published data), though from the article’s point of view, it was only necessary to provide the same conditions for both starch setups.

### Quantification of sterigmatocystin and aflatoxin B_1_ by high performance liquid chromatography

After 3 weeks of incubation, to each flask, 20 mL methanol was added and the whole content of the flask was transferred into a Stomacher bag and pulsified for 45 s in a Pulsifier (Microgen Bioproducts Ltd., Camberley, UK). After 24 h in the dark, the bags were pulsified again for 45 s to finish the extraction. The liquid parts were transferred into 50 mL plastic Falcon tubes and centrifuged at 20 °C, for 10 min, at 3000 rpm. The supernatants were stored at − 20 °C until analysis. Control (blank) samples were extracted, derivatised (AFB1), and analyzed in the same way as for the real samples to determine matrix interferences.

For analytical determination of ST and AFB1 by HPLC with reverse phase chromatography, a modular Shimadzu LC-10AD VP HPLC system (Shimadzu Europa GmbH, Duisburg, Germany) was used, equipped with an SPD-10AVP UV–VIS detector (254 nm) and an RF-20A fluorescence detector for ST and AFB1, respectively. ST was detected by its UV absorption. AFB1 was detected by induced fluorescence.

ST analysis has been performed on a Purospher STAR Rp18e 5 µm 125 × 4 mm column (Merck, Darmstadt, Germany). Five microliters of the methanolic extracts were directly injected into the HPLC system mentioned above. The flow rate of 0.5 mL/min was applied during the separation. Initial eluent composition was 40% of water and 60% of methanol, which was held for 1 min; then, methanol content gradually increased to 80% at 9 min, stayed at this rate for 16 min, and finally decreased to the starting value. External calibration with standard solutions of ST was carried out in the range between 0.010 and 20.0 µg/mL.

AFB1 measurement required derivatisation prior to HPLC-FLD analysis. Dried residues, obtained from 1 mL of each extract were resuspended in 0.4 mL of hexane, and 0.1 mL of trifluoroacetic acid (TFA) was added to form the corresponding derivative at 60 °C for 15 min. Next 0.4 mL of water:acetonitrile (9:1) was added, they were mixed, and the lower (aqueous) phase was collected. Three microliters was injected into HPLC system equipped with a Prodigy C18 150 × 4.6 mm 5 µm column (Phenomenex, Torrance, CA, USA) and fluorescence detector. Isocratic chromatographic separation was applied using an eluent (65:35) containing water and a mixture of methanol:acetonitrile (1:1, v/v%) at a flow rate of 1 mL/min. Fluorescence detection wavelengths of 350 nm and 430 nm were used for the excitation and emission, respectively. Calibration curves were recorded with derivatised AFB1 solutions containing the toxin at levels of 1.25, 2.5, 5, 10, 20, 50, 100, and 200 ng/mL. If the level of the sample was out of the range of the calibration curve, it was tenfold diluted.

Calibration curves, obtained from HPLC peak areas at the corresponding retention times, had excellent linear calibration characteristics for both analytes. The Determination Coefficient values (R^2^) of calibration curves ranged between 0.998 and 1.000, whereas the slopes were 18.3 and 146.5 for AFB1 and ST, respectively. The limits of detection (LODs), defined as analyte concentrations corresponding to the signal-to-noise ratio of 3:1 or greater, were determined with standard solutions. LODs were found to be 1.25 and 10 ng/mL for AFB1 and ST, respectively. Thus, using derivatisation and fluorescence detection mode for AFB1 allowed an order of magnitude improvement in the LOD compared to UV detection (10 ng/mL), in our previous works (Kosztik et al. 2020; Bata-Vidács et al. 2020). The same improved LODs were obtained in spiked liquid matrices extracted from blank samples, indicating no matrix effect under the experimental conditions applied.

### Electron microscopy

A few beads from the rice and corn starch setups at the end of the incubation period were used for electron microscopic studies. The pictures were taken by Evo 40 Zeiss electron microscope (Carl Zeiss SMT Evo Series—SEM Technology, Oberkochen, Germany) at the microscope laboratory of the HUN-REN Hungarian Research Network, Budapest, Hungary. The diameters of the starch granules were determined by the AnalySIS Pro 3.2 software (Soft Imaging System GmbH, Münster, Germany).

## Results

The AFB1 and ST production of *A. flavus* and *A. creber*, respectively, on corn and rice starches were studied to determine whether rice starch is a better substrate for toxin production than corn starch, the same way as rice grain is a tenfold better substrate than corn for this purpose. To properly grow mold capable of producing toxins, a suitable model system using glass beads was developed. The physical and morphological characteristics of corn and rice starches and the mycelial growth on the starch granules mounted on glass beads were studied by scanning electron microscopy.

### Particle size measurements for rice and corn starch

**Fig. 1:**
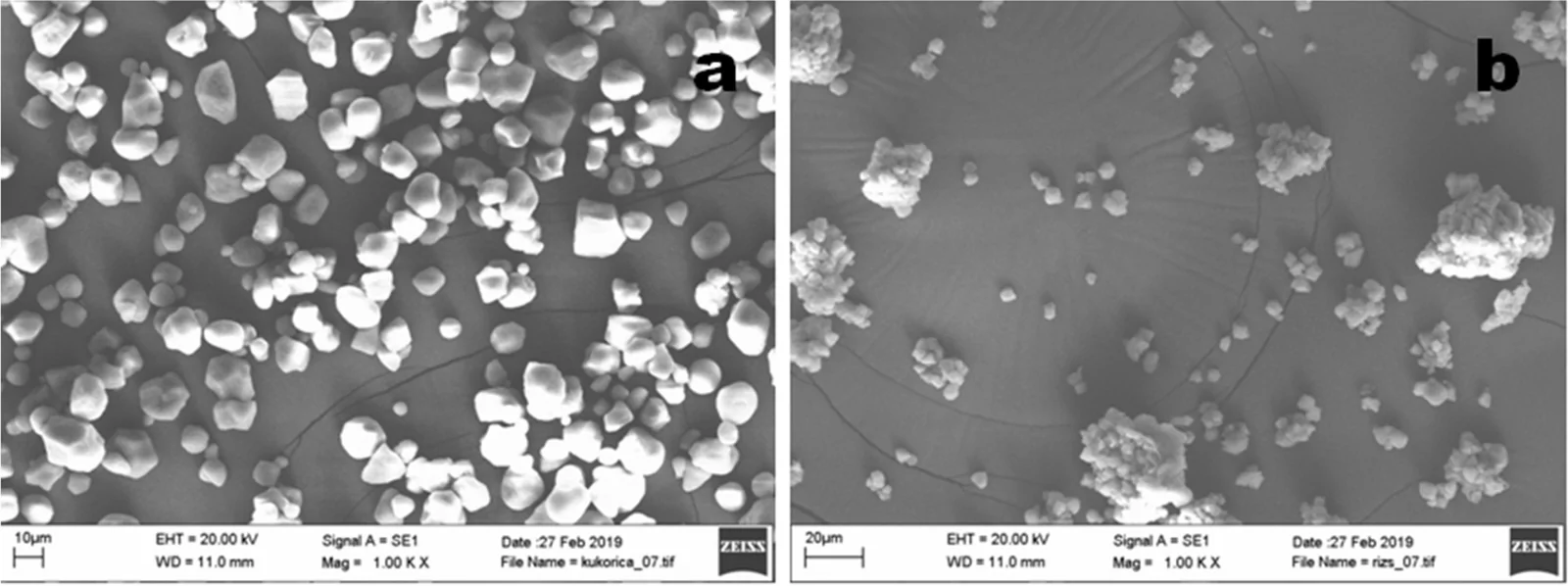
Scanning electron microscopic images of corn (**a**) and rice (**b**) starch particles at × 1000 magnification

**Fig. 2:**
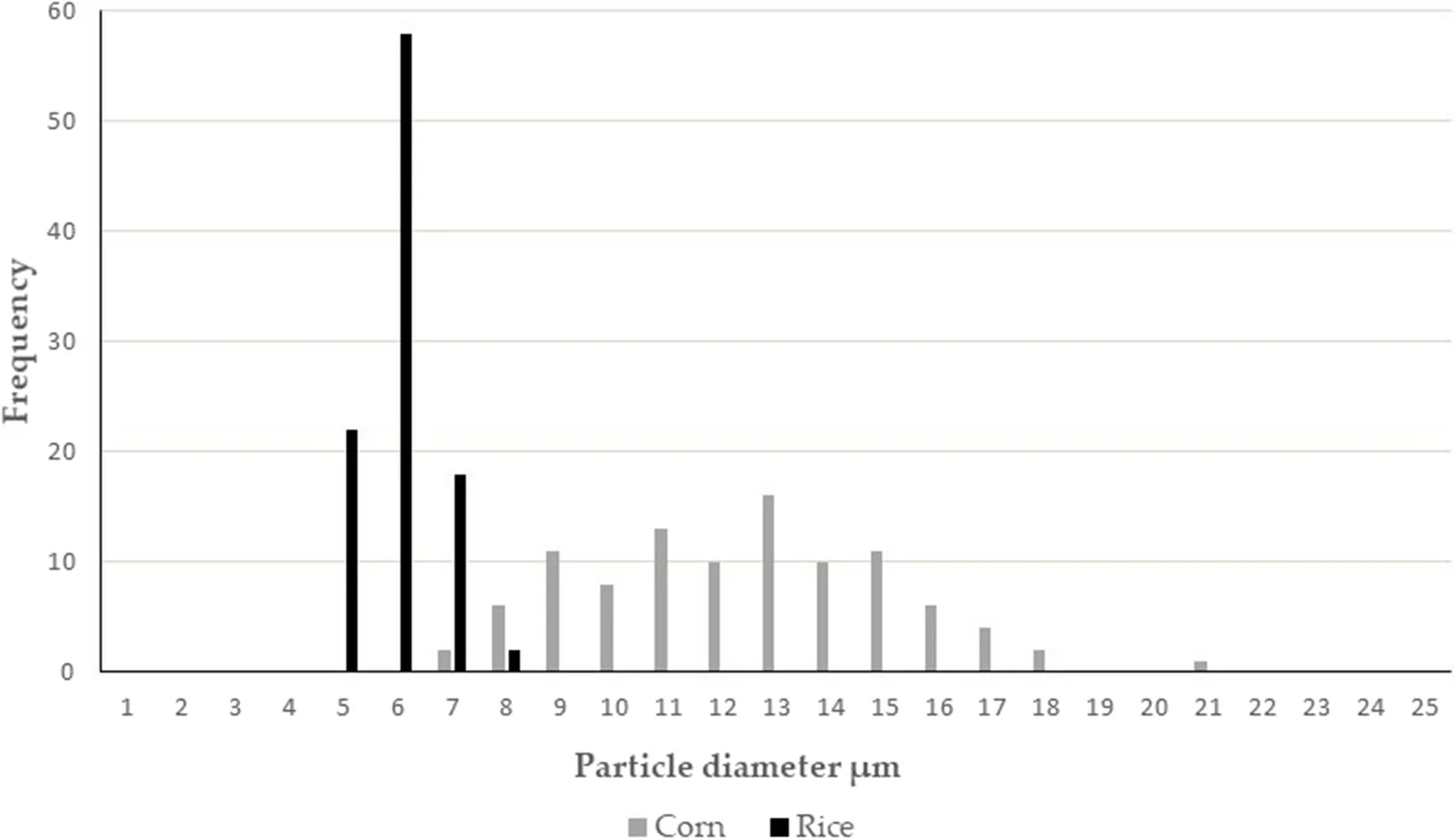
Particle diameter distribution for corn and rice starch granules

The particle size distributions of rice and corn starches were determined from scanning electron microscopy images (Fig. 1). Diameters of a hundred particles of rice and corn starch were measured; and averages, deviations, surfaces, and volumes were calculated. The size distribution is shown in Fig. 2, and other parameters determined are presented in Table 1. According to the results, average particle sizes were 10.9 ± 2.78 µm and 4.54 ± 0.635 µm for corn for rice starch, respectively. These results are similar to the findings of Ali et al. (2016), who reported that the size of the granules varied from 5.2 to 5.9 µm and 11.4–12.0 µm for rice and corn starches, respectively, and also with other studies on rice (Gonzalez and Perez 2002; Simi and Abraham 2008) and corn (Jobling 2004) starches. The morphology of starch granules may be attributed to the physiology and biological origin of the plant and also to the biochemistry of the amyloplast. The amylose and amylopectin ratios might also influence the shape and size of starch particles (Kaur et al. 2007). Structure affects the enzymatic digestibility of starches (Biliaderis 1991). Corn and rice starches showed significant differences regarding morphological and physico-chemical properties. The particle size of corn starch is higher on average compared to the granule sizes of rice starches (Dobolyi et al. 2013).

**Table 1:** Particle parameters for rice and corn starches

	Corn starch	Rice starch
Average particle diameter (µm)	10.90	4.54
Standard deviation	2.780	0.635
Surface (µm^2^)	397	66
Volume (µm^3^)	810.0	51.9
Surface area to volume ratio (1/µm)	0.49	1.27

### Mold growth on corn and rice starches mounted on glass beads

**Fig. 3:**
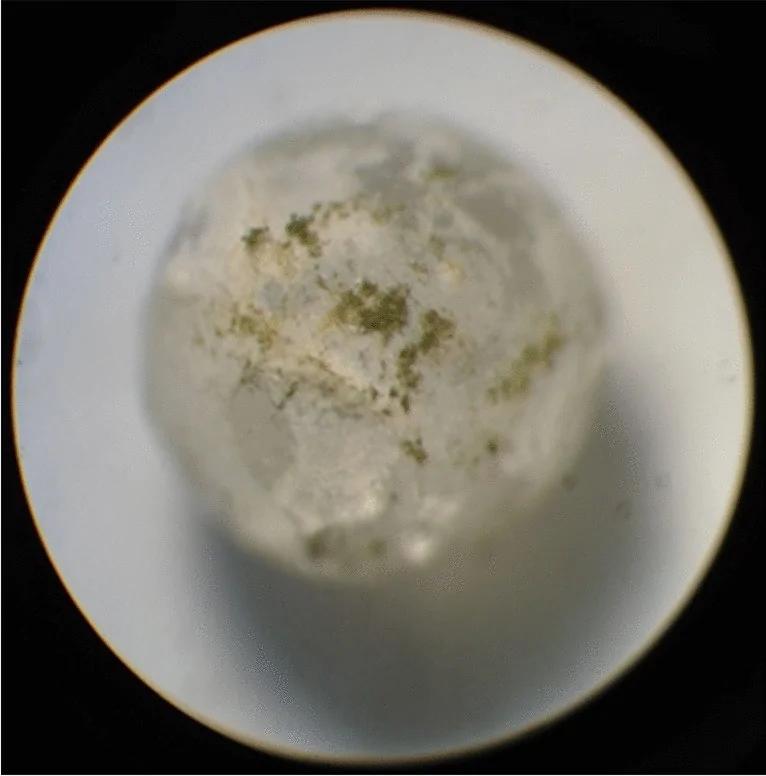
*Aspergillus flavus* mold growth on corn starch mounted on glass bead (× 40 magnification, Zeiss Jena Binocular Stereo Microscope, Germany)

**Fig. 4:**
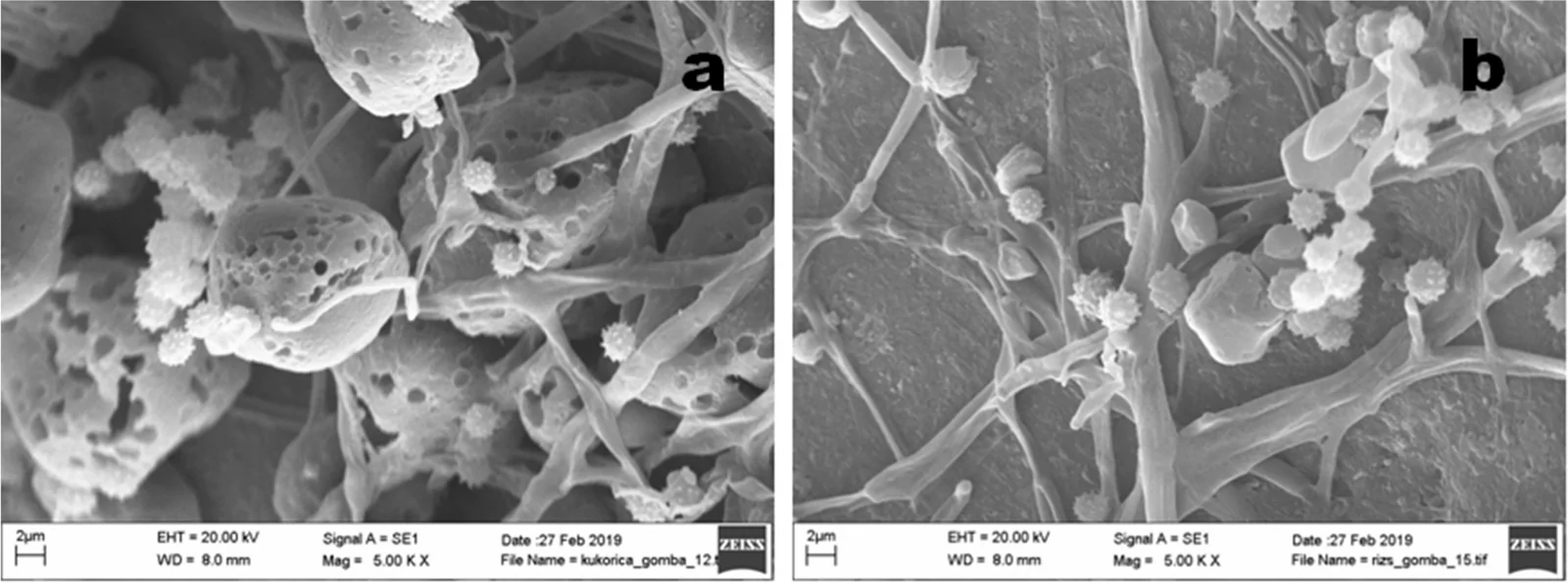
*Aspergillus flavus* Zt41 grown on corn (**a**) and rice (**b**) starch granules mounted on the surface of glass beads (magnification: × 5000). On corn, the sample hydrolysis of starch is seen as holes

A special system was developed with glass beads that can be used with water-absorbing powdery substrates as starch in experiments that model mold growth on the surface of grain pieces, to ensure the free flow of air and the place for mycelial growth between the grains. Glass beads with diameters of 2 mm were chosen to provide similar parameters as corn grit or rice. The starches were mounted on the surface of the beads with adequate amounts of water. The inoculated molds could grow mycelia and even sporulate on the surface as they would on grain grit particles (Figs. 3 and 4).

### Sterigmatocystin production of *A. creber* 2663 and aflatoxin B_1_ production of *A. flavus* Zt41 on corn and rice starches mounted on glass beads

**Fig. 5:**
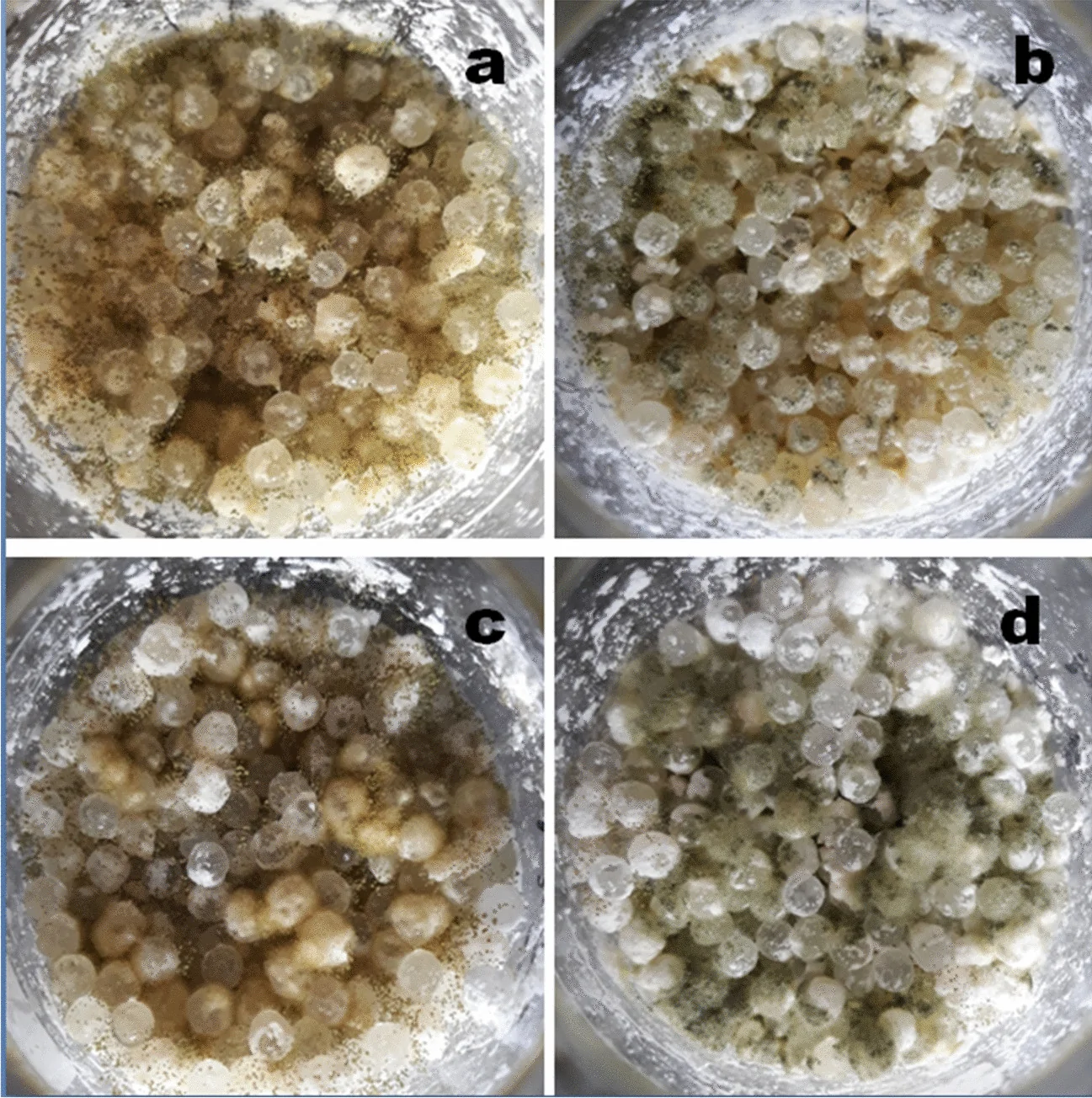
Mold growth on corn and rice starch coated glass beads after 3 weeks of incubation at 26 °C in the dark. *Aspergillus flavus* Zt41 on corn starch (**a**) and on rice starch (**b**), *Aspergillus creber* 2663 on corn starch (**c**) and on rice starch (**d**)

To determine whether rice starch is a better substrate for toxin production than corn starch, rice and corn starches were mounted on glass beads, and the flasks were inoculated with AFB1 producer *A. flavus* Zt41 or ST producer *A. creber* 2663. After 3 weeks of incubation at 26 °C in the dark, both molds on both starches showed good growth and sporulation (Fig. 5).

**Fig. 6:**
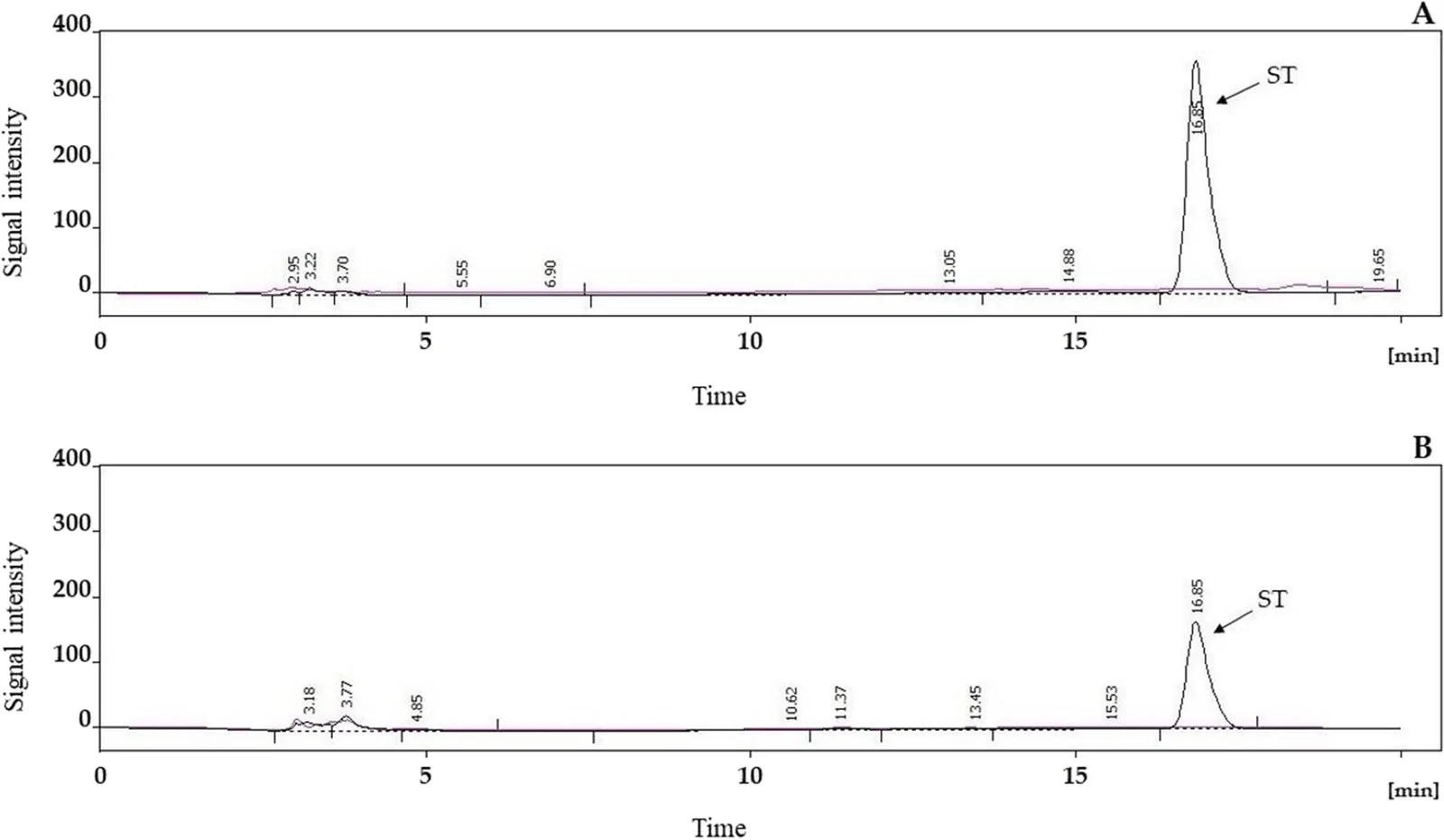
Chromatograms of samples analyzed for sterigmatocystin (ST), extracted blank starch matrix (purple line), and that of containing ST (black line). The upper chromatogram was measured for rice (**A**) and the lower for the corn starch matrix (**B**). Chromatograms were recorded by using UV–VIS detection mode at wavelength 254 nm

**Fig. 7:**
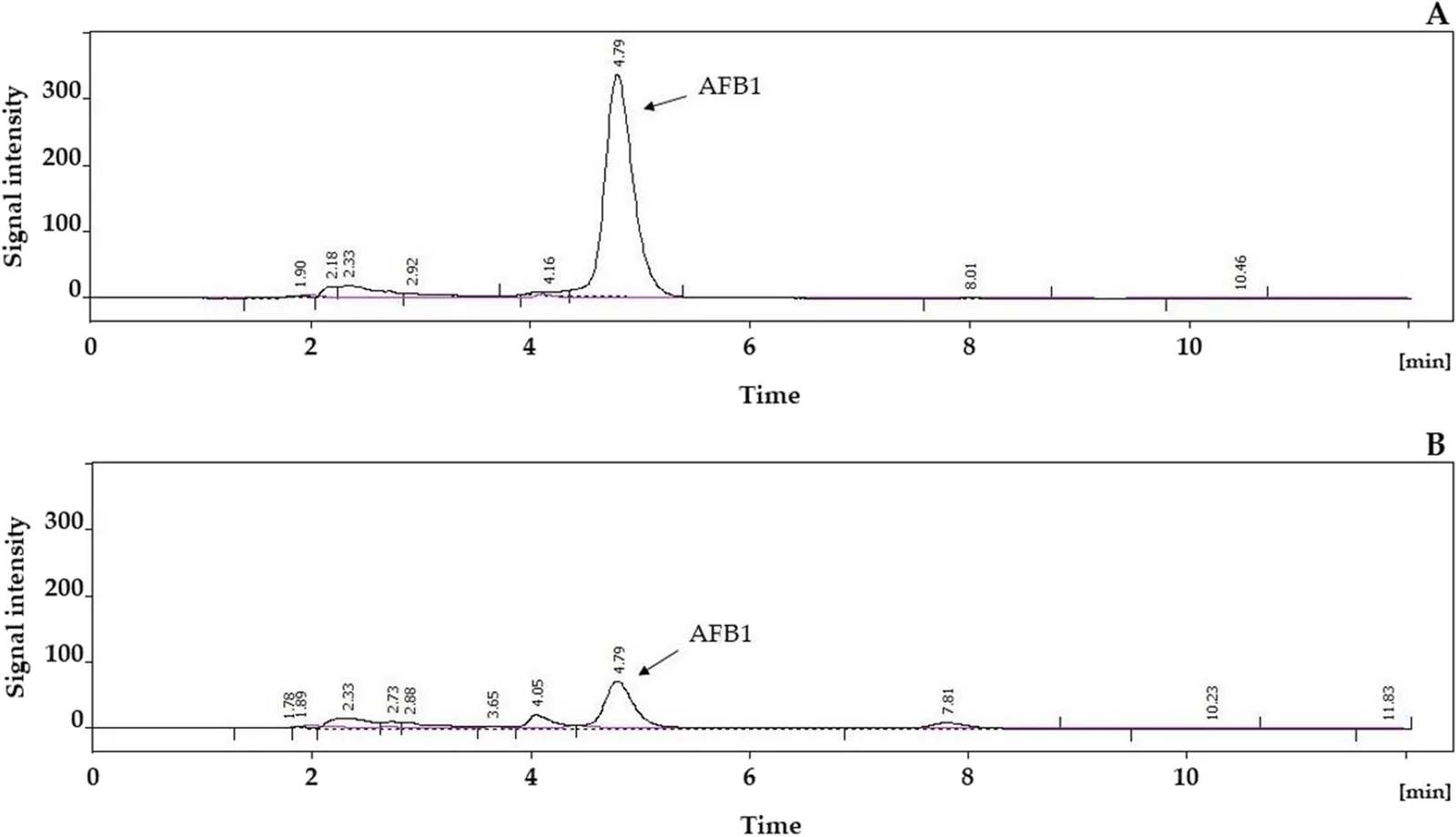
Chromatograms of samples analyzed for aflatoxin B_1_ (AFB1), extracted blank starch matrix (purple line), and that of containing AFB1 (black line). The upper chromatogram was measured for rice (**A**) and the lower for corn (**B**) starch matrix. Chromatograms were recorded by using fluorescence detection mode at wavelengths 350 nm and 430 nm for excitation and emission, respectively

Mycotoxin production in the cultured aspergilli was determined by high performance liquid chromatography (HPLC) using detection by ultraviolet (UV) absorbance of ST and emitted fluorescence of AFB1 derivatised with trifluoroacetic acid (TFA) to form a fluorescent hemiacetal derivative of its terminal fused dihydrofurane ring. Standard chromatograms for ST and AFB1 are shown in Figs. 6 and 7. As blank samples did not contain matrix components, where the target components (ST and AFB1) appear in the chromatograms, quantitation was readily based on instrumental (external) calibration with standard solutions.

AFB1 production of *A. flavus* Zt41 was found to be 437.6 ± 128.4 ng/g and 90.0 ± 44.8 ng/g on rice and corn starch substrates, respectively. *A. creber* 2663 produced 72.8 ± 10.0 µg/g and 26.8 ± 11.6 µg/g ST on rice and corn starch substrates, respectively. A five-fold difference can be observed for AF production between rice and corn starches, and 3 times higher ST production was found on rice starch substrate compared to corn starch.

## Discussion

In our study, we aimed to determine whether the use of high-purity corn and rice starch as the only source of carbon and energy could replicate the large differences in AFB1 and ST production yields observed in corn and rice grains. The developed novel glass bead system effectively simulated the surface-volume ratio parameter range of rice and partially of corn seeds. The surface of the glass beads was coated with starch, and the model molds (*A. flavus* Zt41 and *A. creber* 2663) were able to weave them richly with their mycelial fibers. Electron microscopy images depicted the starch-degrading activity of the mold strains and the process of spore formation. Analytical studies on pure starch substrates also confirmed the empirical fact that mycotoxin production in rice is well above that in corn. A fivefold difference in AFB1 and a three-fold difference in ST production were detected for rice and corn starches. This means that the difference between the toxin production on rice and corn grains is present for rice and corn starches as well. The difference may be due to the different degradability of the different starches, such as the level of the available substrate, maltose, and glucose. The amylose-to-amylopectin ratio has a role in the enzymatic digestibility of a given starch, but this was found to be only a few percent different in in vitro digestibility studies comparing external low-amylose vax rice varieties (You et al. 2014). Since the amylose content of the corn and rice starches used in the experiment was similar, this difference cannot be the main reason for the observed pronounced difference in toxin production. This difference suggests a diversity in metabolic activity, probably due to the ability of *Aspergillus* strains to uptake more metabolizable glucose and maltose from rice starch.

In electron microscopy analysis of starches, we found that rice starch granules are half the diameter of corn starch granules, and thus the surface area to volume ratio for rice starch is more than two and a half times that of corn starch, which can greatly enhance the effectiveness of amylases The smaller size of rice starch granules compared to corn starch provides a larger surface area for the same amount of starch, making nutrients more accessible to molds. A higher degree of hydrolysis of rice starch compared to other starches is mentioned in several publications (Fuwa et al. 1977; Snow and O’Dea 1981), because rice starch with smaller granules has a higher specific surface area, increasing the efficiency of enzymatic hydrolysis.

The increase in hydrolytic activity measured for cellulose with increasing specific surface area is a good analogy for the different degradability of corn and rice starch. Yeh et al. (2010) studied how particle size affected the enzymatic hydrolysis of cellulose. By milling technique, two different types of cellulose were obtained: one with a diameter of about 0.8 µm and a second batch with 2.6 µm. The production rate of cellobiose and glucose increased at least fivefold for the starch with the smaller granules, which the authors explained by the larger specific surface area due to the smaller size.

Considering that the amylose-amylopectin ratios of the starches tested were similar (Garc et al. 2015), and existing literature data suggest that this feature is unlikely to cause such a difference in in vitro digestibility (Jane et al. 1997; Magallanes-Cruz et al. 2017), it is probable that the variance in surface area-to-volume ratio is one of the primary explanations for the higher mycotoxin production on the rice substrate. The developed glass bead system may also be suitable for studying the environmental parameters of metabolites produced by other molds.

## Data Availability

The authors declare that the data supporting the findings of the study are available in the study. If raw data files are required, they are available upon reasonable request from the corresponding author.
